# National Convention for Revising the Pharmacopeia of the U. S.

**Published:** 1849-06

**Authors:** Geo. B. Wooll

**Affiliations:** Vice President of the Convention, 1840; Philadelphia


NATIONAL CONVENTION FOR REVISING THE PHARMACOPEIA OF THE UNITED STATES.

We invite the attention of our readers to the following notice:

The Convention for Revising the Pharmacopeia, which met in Washington in January, 1840, adopted the following resolutions.

1. The President of this Convention shall, on the first day of May, 1849, issue a notice, requesting the several *incorporated State Medi-*

*cal Societies*, the *incorporated Medical Colleges*, the *incorporated Colleges of Physicians and Surgeons*, and the *incorporated Colleges of Pharmacy*, throughout the United States, to elect a number of delegates, not exceeding three, to attend a general convention, to be held at Washington, on the first day of May, 1850.

2. The several incorporated bodies, thus addressed, shall also be requested by the President to submit the Pharmacopeia to a careful revision, and to transmit the result of their labors, through their delegates, or through any other channel, to the next Convention.

3. The several medical and pharmaceutical bodies shall be further requested to transmit to the President of the Convention the names and residences of their respective delegates as soon as they shall have been appointed, a list of whom shall be published, under his authority, for the information of the medical public, in the newspapers and medical journals, in the month of March, 1850.

4. In the event of the death, resignation, or inability to act of the President of the Convention, these duties shall devolve on the Vice President, and, should the Vice President also be prevented from serving, upon the Secretary or Assistant Secretary, the latter acting in the event of the inability of the former.

In compliance with the foregoing resolutions, the undersigned having been informed by the President of the late Convention, Dr. Lewis Condict, that he would be unable, from indisposition, to perform the duties assigned to him, gives notice to the several medical and pharmaceutical bodies enumerated in the first resolution, that a Convention for Revising the National Pharmacopeia will meet in the City of Washington, on the first Monday in May, 1850.

The undersigned also requests of the several bodies referred to, that they will fulfil the wishes of the convention, as set forth in the second resolution; and, further, that they will transmit to his address, on or before the first of March next, the names and residences of the delegates whom they may appoint, in order that a list of them may be published, as directed in the third resolution.

GEO. B. WOOLL, M.D.,
*Vice President of the Convention*, 1840.

Philadelphia, May 1, 1849.

